# Multiples lithiases de l’urètre prostatique associées à une anéjaculation révélant un dysraphisme spinal à type de syndrome de moelle bas attachée chez un jeune homme: à propos d’un cas exceptionnel

**DOI:** 10.11604/pamj.2019.33.165.18092

**Published:** 2019-07-03

**Authors:** Mustapha Ahsaini, Adil Mellouki, Soufiane Mellas, Jalaleddine El Ammari, Mohammed Fadl Tazi, Mohammed Jamal El Fassi, Moulay Hassan Farih

**Affiliations:** 1 Service d’Urologie, Centre Hospitalier Universitaire Hassan II de Fès, Maroc

**Keywords:** Spina bifida, dysraphisme spinale, calcul intra prostatique, syndrome de moelle bas attaché, chirurgie, Spina bifida, spinal dysraphism, intraprostatic stone, tethered cord syndrome at the base of the spinal canal, surgery

## Abstract

Le dysraphisme spinal (ou spina bifida) est une pathologie neurologique qui représente la première cause de handicap urologique congénital. Il peut avoir des expressions cliniques très variables, les troubles vésico-sphinctériens et sexuels sont fréquents et rarement isolés, noyés au sein des troubles moteurs, orthopédiques, sensitifs, digestifs voire cognitifs. Le syndrome de la moelle bas attachée est une complication du dysraphisme spinal. Affection est souvent découverte chez l'enfant. Elle peut être asymptomatique et rencontrée chez l'adulte. Les troubles vésico-sphinctériens constituent la principale cause de morbi-mortalité par les complications uro-nephrologiques qu'ils entrainent avec une altération significative de la qualité de vie de ces patients, justifiant ainsi une prise en charge spécifique, multidisciplinaire et un suivi strict. Nous présentons un cas exceptionnel de dysraphisme spinale type syndrome de la moelle bas attachée de découverte fortuite à l'âge adulte à l'occasion du bilan étiologique de lithiases de l'urètre prostatique associées à une anéjaculation.

## Abstract (en)

Spinal dysraphism (or spina bifida) is a neurological disorder representing the first cause of congenital urological disability. It has several clinical manifestations, vesicosphincteric and sexual disorders are frequent and rarely isolated, belonging to motor disorders, orthopedic, sensory, digestive or even cognitive impairments. Tethered cord syndrome at the base of the spinal canal is a complication of spinal dysraphism. This disorder is often detected in children, may be asymptomatic and found in adults. Bladder sphincter disorders are the main cause of morbi-mortality due to uronephrologic complications with a significant alteration in quality of life. This justifies specific management and multidisciplinary and strict monitoring. We here report an exceptional case of spinal dysraphism such as tethered cord syndrome at the base of the spinal canal found incidentally in an adult patient during an etiological assessment of lithiasis of the prostatic urethra associated with anejaculation .

## Introduction

Le spina bifida (dysraphisme spinal) est une pathologie neurologique congénitale qui regroupe un large spectre de pathologies ayant en commun une anomalie de fermeture du tube neural au stade embryologique, dont environ la moitié est représentée par les dysraphismes fermés (spina bifida occulta), et qui touchent autant les filles que les garçons [1]. Les progrès de la prise en charge notamment des complications vésico-sphinctériennes ont permis d'amener les patients à une espérance de vie quasi normale alors qu'avant 1960, seuls 10% atteignaient l'âge adulte [2]. Les signes cliniques sont peu informatifs du mécanisme physiopathologique et, le fonctionnement vésico-sphinctérien justifie le recours aux explorations urodynamiques sous l'angle d'une approche multidisciplinaire pour une meilleure définition du risque uro-néphrologique, un traitement et un suivi adapté au comportement vésico-sphinctérien ainsi qu'une amélioration de la qualité de vie [2]. Grâce à l'accès à l'imagerie anténatale, la majorité des cas sont diagnostiqués dans la période périnatale ou dans la petite enfance. Selon nos connaissances, notre observation est le premier cas qui illustre un mode de découverte inhabituel, où un dysraphisme spinal fermé a été diagnostiqué chez un jeune homme de 27 ans présentant des signes urinaires obstructifs, avec des lithiases au niveau de l'urètre prostatique, ainsi qu'une anéjaculation responsable d'une stérilité primaire.

## Patient et observation

**Figure 1:**
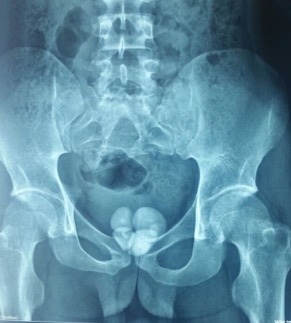
Arbre urinaire sans préparation (AUSP) montrant multiples lithiases centimétriques au niveau de la loge prostatique

**Figure 2:**
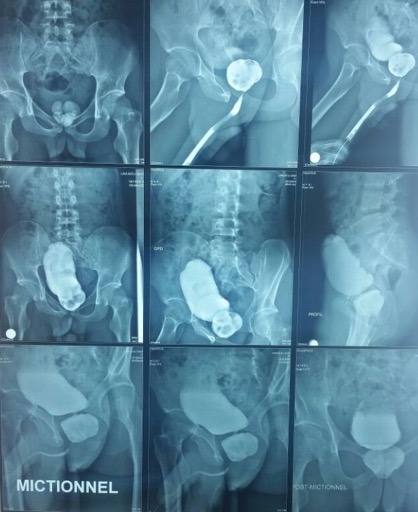
Une UCRM montrant un urètre antérieur perméable, de multiples lithiases centimétriques au niveau de la loge prostatique, une vessie de lutte distendue, et un résidu post mictionnel important

**Figure 3:**
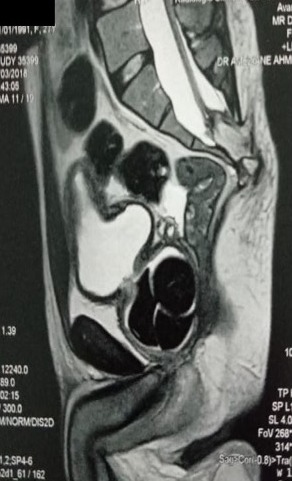
Coupe sagittale d’IRM pelvienne objectivant la présence de cinq formations laminant le parenchyme prostatique (flèche jaune) associé à un dysraphisme spinal fermé avec une moelle bas attachée (flèche rouge)

Il s'agit d'un jeune homme de 27 ans, qui consulte chez son médecin traitant pour des signes urinaires obstructifs, à type de dysurie, de faiblesse du jet urinaire et de sensation de vidange incomplète. Marié depuis 3ans, il présente une stérilité primaire avec une anéjaculation et une dysfonction érectile évoluant depuis cette même période. L'examen clinique révèle une asymétrie du bassin. L’examen neuro-périnéal objective une dépression au niveau de la zone sacrée et au toucher rectal une loge prostatique aplatie et indurée. Il n'existe pas d'anomalies des organes génitaux externes, ni de signes neurologiques sensitivomoteurs. Sa fonction rénale est normale, son étude cytobactériologique des urines est négative. L'uretrocystographie rétrograde et mictionnelle (UCRM) a mis en évidence: à l'arbre urinaire sans préparation (AUSP) de multiples lithiases centimétriques au niveau de la loge prostatique (Figure 1), au temps de remplissage rétrograde: un urètre antérieur perméable, une vessie de lutte distendue, et au temps mictionnel et post-mictionnel: un résidu post mictionnel important sans reflux vésico-urétéral (Figure 2). L'imagerie par résonnance magnétique (IRM) pelvienne a démontré la présence de cinq formations lithiasiques dont la plus grande mesure 4 cm de grand axe laminant le parenchyme prostatique, celui-ci étant réduit à une fine lame de tissu. Elle a également objectivé la découverte d'un dysraphisme spinal fermé associant une moelle bas attachée, un rachischisis, accompagné d'une dysmorphie du sacrum (Figure 3).

**Figure 4:**
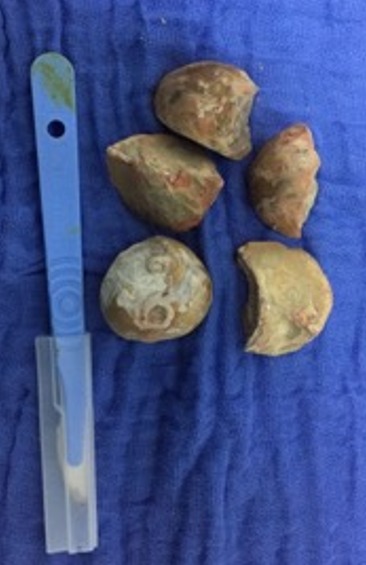
Extraction de 5 pièces lithiasiques par cystolithotomie type Freyer

Le bilan hormonal du patient comprenant la *Follicle Stimulating Hormone* (FSH), la *Luteinizing Hormone* (LH) et la testostérone s'inscrit tout à fait dans les normes. Toutefois, le spermocytogramme ne fut pas réalisé vu l'impossibilité de recueil de sperme secondaire à l'anéjaculation évoluant depuis déjà 2 ans, de même la recherche de spermatozoïdes dans les urines est infructueuse. Le patient a bénéficié d'une cystolithotomie type Freyer pour extraction des calculs de l'urètre prostatique (Figure 4). Les suites opératoires étaient simples, une sortie à domicile a été envisagée à J+3 et une ablation de la sonde vésicale a été réalisée à J+7. Le patient a été vu régulièrement lors des consultations de contrôle, il a aussi bénéficié deux mois après d'un bilan urodynamique visant une meilleure caractérisation du fonctionnement vésico-sphinctérien. Celui-ci a mis en évidence une hyperactivité détrusorienne, une hypertonie sphinctérienne et une dyssynérgie vésico-sphinctérienne avec une compliance vésicale normale. L'association du sondage intermittent aux anticholinergiques a été préconisé dans ce cas, le patient a d'ailleurs fait preuve d'une bonne adhésion au traitement. On note également une bonne amélioration de la fonction érectile sous inhibiteur de la phosphodiestérase de type 5 (IPDE-5), néanmoins, aucun changement n'est à signaler concernant la fonction éjaculatoire et une prise en charge dans le cadre d'une procédure de procréation médicale assisté (PMA) a été envisagée chez notre patient. Enfin et après avis de notre équipe neurochirurgicale aucun traitement chirurgical ne peut être proposé à notre patient à cet âge vu le risque élevé de complication en dépit de gain aléatoire.

## Discussion

Le spina bifida (ou dysraphisme spinal) est une pathologie neurologique qui représente la première cause de handicap urologique congénital. Il comprend un spectre hétérogène de malformations ayant en commun une même origine embryologique par défaut plus ou moins important de fermeture du tube neural; on les distingue classiquement en dysraphismes ouverts (myéloméningocèle) et fermés [1]. Au Maroc, la prévalence moyenne des anomalies de fermetures du tube neural parmi les naissances vivantes avait passé de 21,78 en 2008 à 12,1 cas par 10000 naissances vivantes en 2011 [3]. Cette incidence est en diminution dans les pays développés du fait premièrement de l'efficacité de la prévention par acide folique [4] et grâce à l'accès à l'imagerie anténatale et au droit à d'interruption médicale de grossesse. Ils peuvent avoir des expressions cliniques très variables, mais les troubles vésico-sphinctériens sont rarement isolés, noyés au sein des troubles moteurs, orthopédiques, sexuels, sensitifs, digestifs voire cognitifs [4]. Sur le plan clinique, l'ensemble des signes urinaires peuvent être présents (rétention, syndrome d'hyperactivité vésicale, incontinence par impériosités ou à l'effort) selon le type d'atteinte vésicale et sphinctérienne [5]. Concernant les troubles sexuels, la dysfonction érectile et la dysfonction éjaculatoire, sont plus fréquentes que chez la population générale [6], ce qui fait la particularité de notre observation c'est son mode de révélation particulier où juste les troubles. Le retentissement sur la qualité de vie semble important, d'autant plus que les autres déficiences et limitations d'activité sont modérées, mais difficilement évaluable, il est mieux authentifié en ce qui concerne l'estime de soi, vitalité, et projets familiaux [5]. Sur les plans urodynamique et physiopathologique, l'hétérogénéité est également de mise, puisque l'ensemble des combinaisons entre hypo et hyper activité sphinctérienne ou détrusorienne peuvent se rencontrer [1, 4, 6]. Les signes cliniques sont peu informatifs sur les mécanismes urodynamiques, justifiant ainsi le recours systématique aux explorations urodynamiques afin de déterminer le type de fonctionnement vésico-sphinctérien du patient et donc le niveau de risque pour le haut appareil. Elle permet également de guider et d'évaluer les prises en charge thérapeutiques qu'elles soient médicamenteuses ou chirurgicales [2, 6]. L'urodynamique est donc une constante dans le suivi des patients atteints de spina bifida mais ne peut se concevoir que dans une approche pluridisciplinaire tant les déficiences associées peuvent être nombreuses, dans le but de donner au patient les meilleures autonomie, participation et qualité de vie tout en préservant le haut appareil urinaire [2]. Les tableaux urodynamiques les plus fréquents sont [5, 6]: hyperactivité détrusorienne et hypertonie sphinctérienne; hypoactivité détrusorienne et hypertonie sphinctérienne; hyperactivité détrusorienne et hypoactivité sphinctérienne; hypoactivité détrusorienne et hypoactivité sphinctérienne. L'association d'une hyperactivité vésicale et d'une hypertonie sphinctérienne avec dyssynérgie est la plus fréquente et la plus délétère pour le haut appareil, c'est le cas de notre patient [1, 7]. Les troubles vésico-sphinctériens ont été longtemps la cause principale de morbi-mortalité après la période néonatale par les complications qu'ils entrainent sur le haut appareil urinaire notamment (4,5) [2, 4]. Le reflux vésico-urétéral, causé par un régime d'hyperpression vésicale (notamment quand les pressions vésicales s'élèvent au-dessus de 40cm H_2_O), peut être à l'origine d'une hydronéphrose et à terme d'une insuffisance rénale chronique dont le risque est huit fois plus élevé que dans la population générale [1, 6]. Les infections urinaires hautes à répétition sont d'autre part source de cicatrices rénales participant à la réduction néphronique [2]. La pathologie lithiasique est plus fréquente que dans la population générale surtout après agrandissement vésical [7], et les cancers de vessie ne semblent pas plus fréquent mais surviennent chez des patients plus jeunes, sont plus invasifs et les carcinomes épidermoïdes prédominent. Enfin, la fréquence de l'allergie au latex chez ces patients doit faire exclure ce matériau des différentes prises en charge à partir de la période néonatale [2, 4]. La prise en charge neuro-urologique du patient spina bifida doit avoir pour objectifs la prévention des complications sur le haut appareil urinaire en assurant un remplissage de la vessie à basse pression et une vidange complète, régulière et à basse pression, et d'autre part un objectif fonctionnel en assurant une continence urinaire et fécale, le tout dans l'optique d'une meilleure autonomie gage d'intégration sociale et de meilleures qualité de vie et estime de soi [2, 6]. Le traitement « étiologique » neurochirurgical notamment des interventions de libération médullaire pour le syndrome d'une attache basse de la moelle épinière semble avoir une importance surtout, quand il est réalisé précocement [8]. Les effets du traitement sont discutés, mais aucune étude contrôlée randomisée n'a été réalisée jusqu'a lors.

La prise en charge médicale est désormais le plus souvent instituée dès la naissance par anticholinergique et sondages intermittents et régulièrement adaptée aux données urodynamiques, morphologiques et biologiques [1, 2]. Sur le plan médicamenteux, les anticholinergiques ont montré leur efficacité clinique et urodynamique (augmentation de la capacité vésicale et réduction des pressions vésicales) chez l'enfant comme chez l'adulte, en utilisant si nécessaire de fortes doses [6]. Les injections intradétrusoriennes de toxine botulique A représentent une avancée par leur résultats cliniques et urodynamiques probants et en permettant de surseoir ou de décaler une prise en charge non conservatrice [6]. Le sondage urinaire intermittent est le mode mictionnel à privilégier. Il nécessite un apprentissage et la prise en compte des autres déficiences de la maladie, au mieux de manière pluridisciplinaire dans les cas complexes. Concernant les techniques de stimulation nerveuse périphérique, la littérature est peu prolixe mais la neuromodulation sacrée semble prometteuse [9]. Notre patient a bénéficié d'un traitement médical par des anticholinergiques pour son hyperactivité vésicale, associé à un sondage intermittent pour palier au problème de l'hypertonie sphinctérienne. Les traitements chirurgicaux viennent en derniers recours après échec des mesures conservatrices. L'agrandissement vésical, le plus souvent par entérocystoplastie, est un traitement efficace et durable de l'hyperactivité vésicale avec hypocompliance, au prix de complications le plus souvent modérées. Cela nécessite pour le patient de pouvoir réaliser des autosondages intermittents [1, 5]. La prise en charge des complications secondaires de cette affection notamment les complications lithiasiques, comme le cas de nôtre patient qui présente de multiples calculs intraprostatique dont la masse lithiasique > à 5 cm, ou la chirurgie ouverte constitue une vrai alternative chirurgicale en réalisant une prostatolithotomie transvésicale selon technique de Freyer avec incision du col vésical et ablation des calculs intraprostatique et reconstruction du col. D'autres alternatives thérapeutiques peuvent être proposé notamment une prostatolithotomie rétropubienne selon la technique de Millin [10], tout en signalant la difficulté de la réalisation du traitement endoscopique vue la taille et le nombre des calculs. Concernant la prise en charge des complications sexuelles notamment l'anéjaculation due à l'hypotrophie du parenchyme prostatique avec un retentissement sur la fertilité ce qui explique le recours à l'assistance médicale à la procréation (AMP) comme le cas de nôtre patient, par rapport à la prise en charge de la dysfonction érectile, un traitement médical à base IPDE-5 permis le plus souvent une bonne amélioration de la qualité des rapports sexuelles comme le cas chez notre jeune patient. Les patients avec dysraphisme spinal fermé nécessitent pour tous les aspects précédents une surveillance régulière clinique, morphologique, urodynamique et biologique. En 2007, le Groupe d´Études de Neuro-Urologie de Langue Française (GENULF) [11] a proposé des recommandations de suivi communes au blessé médullaire et au spina bifida. L'évaluation de base comporte une consultation de neurourologie aidée d'un calendrier mictionnel, un examen urodynamique, une mesure de la clairance de la créatinine sur urines des 24 heures et une échographie rénale. La surveillance ultérieure dépend du statut à risque ou non sur le haut appareil, au minimum à un rythme annuel chez les patients à risque [11].

## Conclusion

Le syndrome de la moelle bas attachée est une complication du dysraphisme spinal. Affection est souvent découverte chez l'enfant. Elle peut être asymptomatique et rencontrée chez l'adulte. La lithiase de l'urètre prostatique peut être un mode de révélation exceptionnel du syndrome de la moelle bas attachée. Les troubles vésico-sphinctériens ont longtemps été longtemps la cause principale de morbi-mortalité après la période néonatale par les complications qu'ils entrainent sur le haut appareil urinaire. Les progrès de la prise en charge notamment des complications vésico-sphinctériennes, ont permis d'amener les patients à une espérance de vie quasi normale. Le traitement neurochirurgical précoce et préventif semble avoir de meilleurs résultats mais il ne fait pas l'unanimité jusqu'a lors.

## Conflits d’intérêts

Les auteurs ne déclarent aucun conflit d'intérêts.
